# The stratification of patient risk depending on the size and ratio of metastatic lymph nodes in papillary thyroid carcinoma

**DOI:** 10.1186/s12957-017-1141-4

**Published:** 2017-04-04

**Authors:** Young Ran Hong, So Hee Lee, Dong Jun Lim, Min Hee Kim, Chan Kwon Jung, Byung Joo Chae, Byung Joo Song, Ja Seong Bae

**Affiliations:** 1 Department of Surgery, CHA Bundang Medical Center, CHA University, Gyeonggi-do, Korea; 2 Department of Surgery, Seoul St. Mary’s Hospital, College of Medicine, The Catholic University of Korea, Seoul, Republic of Korea; 3 Department of Internal Medicine, Seoul St. Mary’s Hospital, College of Medicine, The Catholic University of Korea, Seoul, Republic of Korea; 4 Department of Hospital Pathology, Seoul St. Mary’s Hospital, College of Medicine, The Catholic University of Korea, Seoul, Republic of Korea

**Keywords:** Risk stratification, Prognosis, Lymph node size, Ratio of lymph node, Papillary thyroid carcinoma

## Abstract

**Background:**

The aims of this study were to identify the clinical significances of the size of metastatic lymph node (mLN) and LN ratio (LNR) and to attempt to create a risk stratification for papillary thyroid carcinoma (PTC) patients.

**Methods:**

We investigated the 435 PTC patients who underwent radioactive iodine (RAI) ablation treatment following thyroid surgery. The patients were classified into two groups (micrometastasis (pN1mic) ≤ 0.2 cm and macrometastasis (pN1mac) > 0.2 cm) and were stratified into the following three risk groups: group I (pN1mic, LNR ≤ 0.5); group II (pN1mic, LNR > 0.5 or pN1mac, LNR ≤ 0.5); and group III (pN1mac with LNR > 0.5). And then we investigated the association of the classified groups and variable clinicopathologic factors.

**Results:**

The clinical characteristics such as large tumor size, extrathyroidal extension, higher T stage, and greater number of mLN or LNR were significantly associated with pN1mac. The mean stimulated thyroglobulin levels were increased with the patient risk groups (*p* = 0.02). The recurrence-free survivals were significantly different between the stratified groups (*p* = 0.001).

**Conclusions:**

The patient groups I, II, and III may be referred to as low-, intermediate-, and high-risk groups. Clinicians should consider the possibility of recurrence, and the decisions about the application of RAI ablation based on the size of mLN and the patient’s risk groups.

## Background

Lymph node metastasis (LNM) occurs in approximately 20-90% of papillary thyroid carcinoma (PTC) cases [1, 2]. LNM is a reliably predictive factor of recurrence and prognosis in thyroid malignancy. Additionally, the size of the primary tumor, the tumor stage, the presence of extrathyroidal extension (ETE), the number of metastatic LNs (mLNs), the size of mLNs, and the ratio of mLNs are known to be correlated with recurrence or prognosis [3–5]. In a recent study, the American Thyroid Association (ATA) risk category and stimulated thyroglobulin (sTg) level were highlighted as predictors of recurrence or survival in patients with PTC following surgery, which suggests that patients with these risk factors should be carefully follow-up [6–8].

Patients with PTC with LNM are subjected to additional radioactive iodine (RAI) ablation treatment after surgery to reduce the rate of relapse. However, it is questionable whether RAI ablation should be applied to all patients with LNM. Furthermore, the benefits and indications for RAI ablation treatment have not been fully elucidated. Importantly, RAI ablation should be performed after the proper selection of patients to minimize the toxicity of the treatment and to maintain the quality of life [9, 10].

We attempted to perform a risk stratification based on the mLN status following the recognition of nodal metastasis as an important prognostic factor. Specifically, the risk of recurrence was expected to be higher among the high-risk patients who satisfied the conditions of larger mLNs or higher lymph node ratio (LNR) [11–13]. However, the overall significance of the size of mLN in PTC has not been extensively studied.

The present study was designed to identify the clinicopathologic factors that are significantly associated with the size of mLN and LNR and to attempt to create a risk stratification based on the size of mLN and LNR for patients with PTC.

## Methods

### Patients

This study is a retrospective review of a prospectively collected database of patients who underwent RAI ablation treatment following thyroid surgery. All patients were free of distant metastasis of the thyroid carcinoma and suspicious malignancy or malignancy of thyroid based on preoperative fine-needle aspirational biopsy. The patients were offered total thyroidectomy with central lymph node dissection (CLND, unilateral, or bilateral) or modified radical neck node dissection (MRND, unilateral, or bilateral) between April 2012 and December 2014 according to our standard institutional protocols that are based on the ATA treatment guidelines [14].

The patients who met the following criteria were excluded from the study: (1) patients with MRND (*n* = 145) including the patients who had undergone only MRND without thyroidectomy due to the recurrence of the disease (*n* = 66); (2) patients for whom PTC had not been confirmed by postoperative final pathology results (*n* = 93); (3) patients who had been confirmed to be negative for nodal metastasis or had not undergone RAI ablation for any reason (*n* = 1445); (4) patients with insufficient data and those without radiologic or pathologic evaluations; and (5) patients lost to follow-up and those with missing data.

**Fig. 1:**
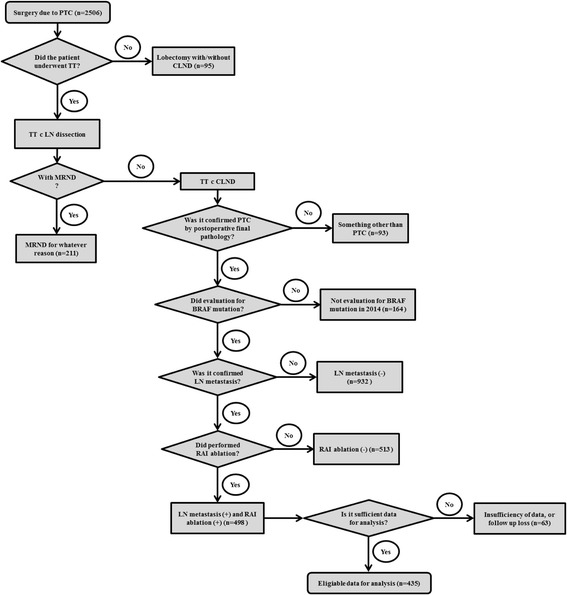
The schematic diagram showed a process to choose the eligible patient for analysis

Consequently, a total of 435 patients were eligible for the analysis (Fig. 1). Our study was approved by the institutional review board (KC15RISI0455).

## Management

### Surgical treatment

All patients were evaluated with a radiologic examination, such as thyroid ultrasonography or enhanced neck computed tomography (CT), prior to surgery and were subjected to an additional biopsy if there was a suspicion of lateral neck LN metastasis.

All surgeries were performed by a single experienced endocrine surgeon. All eligible patients underwent total thyroidectomy with prophylactic or therapeutic CLND. CLND was performed by systematic removal of the LNs in the central compartment (level VI). The proportion of prophylactic CLND was 83.9% (365/435), and the proportion of therapeutic CLND was 16.1% (70/435) that the 435 patients except 145 patients who underwent the MRND.

### Histopathological examination

We received the final pathology results from a single experienced pathologist. All specimens were examined for the primary tumor size, multifocality, the PTC variant, ETE, the total number of the harvested LNs and mLNs, the size of mLN, and BRAF mutations. Specifically, all metastatic LN foci were microscopically measured; in cases of multiple metastatic foci, the largest was recorded as the size of mLN.

### RAI ablation

The criteria for RAI ablation treatment were the following: (1) a tumor size greater than 1 cm regardless of node metastasis, and (2) node metastasis regardless of tumor size. In some cases, the treatment was not performed because the patient refused RAI ablation.

The patients underwent one of two methods of thyrotropin stimulation based on their preference: the administration of recombinant human thyrotropin (TSHalfa, Thyrogen®; Genzyme, Cambridge, MA), or thyroid hormone withdrawal. RAI ablation was conducted within approximately 3 months after the surgery, and the sTg level was measured at the time of this treatment. Stimulation Tg was defined as a serum Tg level that was measured when the serum thyrotrophin concentration was > 30 mIU/L due to either recombinant thyrotropin injections or four-week thyroid hormone withdrawal. Our standard dose was 100 mCi I^131^ for the treatment of thyroid cancer.

## Definitions

The LNR was defined as the total number of mLNs divided by the total number of the harvested LNs.

Also, minimally ETE (mETE) and grossly ETE (gETE) were separately defined within if the ETE was presented. mETE indicated only the presence microscopic capsular invasion, and gETE was defined by the macroscopic involvement of more than a strap muscle including T4.

The recurrence of disease was defined by a confirmed recurrent tumor at a loco-regional and/or distant site by pathological or radiological evaluation.

## Statistics

We used chi-squared tests or *t* tests to compare categorical and continuous variables between the two groups. Similar statistics were applied to the three stratified groups. The results are described as the means ± the standard deviations (SDs) as numbers (%) and were considered statistically significant at *p* < 0.05. A multivariate binary logistic regression model was used to analyze the variables that were found to be statistically significant in the univariate analyses. The results are represented as odds ratios (ORs) with the 95% confidence intervals (CIs). We conducted a receiver operating characteristic (ROC) curve analysis to identify a LNR cutoff value. Recurrence-free survival (RFS) was compared between the patient groups using the Kaplan-Meier method and log rank tests with time intervals that were calculated as months from the date of operation to the date of recurrence or last follow-up.

## Results

### Clinicopathologic features according to the size of mLN

A total 435 patients with a mean age of 46 years (±12 years) old were included. The proportion of females was 72.6%. The patients were classified into two groups (micrometastasis (pN1mic) ≤ 0.2 cm, *n* = 198 and macrometastasis (pN1mac) > 0.2 cm, *n* = 237) according to the size of mLN for the analyses [15].

Comparison of the postoperative pathologic results revealed several factors that were significantly different between the two groups. A larger size of mLN was related to aggressive characteristics, such as a larger primary tumor size, the presence of grossly ETE, and a higher T stage. ETE was confirmed in 60.0% of all patients, and gETE was identified in a significantly higher proportion of the pN1mac group (11 patients, 9.9% versus 28 patients, 18.7%; *p* = 0.05). Additionally, the mean number of mLN was different between the two groups (*p* < 0.001).

We have conducted a ROC curve analysis to identify a LNR cutoff value that predicted disease recurrence with highest sensitivity and specificity. The result was showed as the sensitivity/specificity of LNR >0.5 was 67.9%/65.4%, respectively (area under ROC curve, 0.663; SE, 0.075; *p* = .005).

The mean numbers of metastatic LNs and LNR were also significantly different between the two groups. In contrast, the PTC variant, multifocality, and BRAF mutation were not apparently relevant to the size of the mLN (Table 1).

**Table 1:** Univariate analysis of clinicopathological characteristics on the size of mLN

Characteristics	pN1mic (*n* = 198)	pN1mac (*n* = 237)	*p*
Gender			0.183
Male (27.4%)	48 (24.2%)	71 (30.0%)	
Female (72.6%)	150 (75.8%)	166 (70.0%)	
Age of diagnosis (years)			
Mean age (±SD), year	46.5 (±11.5)	45.4 (±12.1)	0.104
<45 years	88 (44.4%)	113 (47.7%)	0.5
≥45 years	110 (55.6%)	124 (52.3%)	
Primary tumor size			
Mean size (±SD), cm	1.04 (±0.7)	1.38 (±0.9)	0.03
≤1 cm	132 (66.7%)	132 (55.7%)	0.02
>1 cm	66 (33.3%)	105 (44.3%)	
PTC variant			0.066
Classic	148 (74.7%)	196 (82.7%)	
Follicular v.	15 (7.6%)	8 (3.4%)	
Tall cell v.	20 (10.1%)	13 (5.5%)	
Diffuse sclerosing v.	1 (0.5%)	4 (1.7%)	
Others	14 (7.1%)	16 (6.8%)	
Multifocality			0.306
(−)	89 (44.9%)	95 (40.1%)	
(+)	109 (55.1%)	142 (59.9%)	
Extrathyroidal extension			0.125
Absent (40.0%)	87 (43.9%)	87 (36.7%)	
Present (60.0%)	111 (56.1%)	150 (63.3%)	0.05
Minimally	100 (90.1%)	122 (81.3%)	
Grossly	11 (9.9%)	28 (18.7%)	
Mean number of mLN	2.21 (±1.9)	5.47 (±4.5)	<0.001
L/N ratio			<0.001
≤0.5 (78.9%)	174 (87.9%)	169 (71.3%)	
>0.5 (21.1%)	24 (12.1%)	68 (28.7%)	
T stage			0.009
T1a	90 (45.5%)	75 (31.6%)	
T2	2 (1.0%)	10 (4.2%)	
T3	105 (53.0%)	151 (63.7%)	
T4a	1 (0.5%)	1 (0.4%)	
BRAF mutation			0.15
Absent (12.6%)	30 (15.2%)	25 (10.5%)	
Present (87.4%)	168 (84.8%)	212 (89.5%)	
Stimulation Tg			
Mean sTg (±SD), ng/ml	1.27 (±4.8)	2.54 (±8.9)	0.02
<1 ng/ml	158 (79.8%)	167 (70.5%)	0.026
≥1 ng/ml	40 (20.2%)	70 (29.5%)	

*Abbreviations*: *LN* lymph node, *Tg* thyroglobulin, L/N ratio, metastatic LNs/ harvested LNs

The mean sTg value of the pN1mac group was higher than that of the pN1mic group (1.27 ng/ml versus 2.54 ng/ml, *p* = 0.02). Furthermore, a significant result was obtained when a cutoff value of 1 ng/ml was applied to the sTg level (*p* = 0.026).

In the multivariate analysis, the presence of gETE and the m-LN ratio of >0.5 were significantly associated with macrometastasis (OR 2.375, *p* = 0.025, and OR 2.748, *p* = 0.003, respectively; Table 2).

**Table 2:** Multivariate analysis of clinicopathological characteristics on the size of mLN

Characteristics	Odds ratio	95% CI	*p*
Primary tumor size (cm) (≤1 cm versus >1 cm)	0.790	0.466 to 1.341	0.383
ETE (minimally versus grossly)	2.375	1.114 to 5.061	0.025
mLN ratio (≤0.5 versus >0.5)	2.748	1.400 to 5.395	0.003

*Abbreviations*: *LN* lymph node, *ETE* extrathyroidal extension, LN ratio, metastatic LNs/ harvested LNs

### Clinicopathologic features according to patient risk group

We created a three-group risk stratification based on the size of mLN and LNR (cutoff: 0.5) as follows: group I, pN1mic with LNR ≤ 0.5; group II, pN1mic with LNR > 0.5 or pN1mac with LNR ≤ 0.5; and group III, pN1mac with LNR > 0.5.

The univariate analyses revealed that the group III contained more males (42.6%) and was younger (mean age 40.3 years) than the other two groups. Moreover, the univariate analyses also indicated that the factors that were significantly different between the three groups included the primary tumor size (*p* = 0.03), the PTC variant (*p* = 0.04), the presence of ETE (*p* = 0.001), the mean numbers of mLNs (*p* = 0.001), the T stage (*p* = 0.035), and the sTg level (*p* = 0.001) (Table 3).

**Table 3:** Univariate analysis of clinicopathological characteristics on the patient’s risk groups

Characteristics	Group I (*n* = 174)	Group II (*n* = 193)	Group III (*n* = 68)	*p*
Gender				0.009
Male (27.4%)	43 (24.7%)	47 (24.4%)	29 (42.6%)	
Female (72.6%)	131 (75.3%)	146 (75.6%)	39 (57.4%)	
Age of diagnosis (years)				
Mean age (±SD), year	46.7 (±12.0)	46.5 (±12.2)	40.3 (±11.3)	0.001
<45 years	75 (43.1%)	81 (42.0%)	45 (66.2%)	0.002
≥45 years	99 (56.9%)	112 (58.0%)	23 (33.8%)	
Primary tumor size				
Mean size (±SD), cm	1.02 (±0.73)	1.30 (±1.07)	1.58 (±1.47)	0.03
≤1 cm	114 (65.5%)	117 (60.6%)	33 (48.5%)	0.052
>1 cm	60 (34.5%)	76 (39.4%)	35 (51.5%)	
PTC variant				0.04
Classic	128 (73.6%)	162 (83.9%)	54 (79.4%)	
Follicular v.	14 (8.0%)	9 (4.7%)	0 (0%)	
Tall cell v.	19 (10.9%)	9 (4.7%)	5 (7.4%)	
Diffuse sclerosing v.	1 (0.6%)	2 (1.0%)	2 (2.9%)	
Others	12 (6.9%)	11 (5.7%)	7 (10.3%)	
Multiplicity				0.429
(−)	78 (44.8%)	75 (38.9%)	31 (45.6%)	
(+)	96 (55.2%)	118 (61.1%)	37 (54.4%)	
Extrathyroidal extension				0.001
Absent (35.3%)	77 (44.3%)	69 (35.8%)	28 (41.2%)	
Present (64.7%)	97 (55.7%)	124 (64.2%)	40 (58.8%)	
Minimally	86 (88.7%)	100 (80.6%)	36 (90.0%)	0.161
Grossly	11 (11.3%)	24 (19.4%)	4 (10.0%)	
Mean number of mLN	1.88 (±1.12)	5.25 (±4.81)	8.12 (±4.51)	0.001
T stage				0.035
T1	79 (45.4%)	63 (32.6%)	23 (33.8%)	
T2	2 (1.1%)	5 (2.6%)	5 (7.4%)	
T3	92 (52.9%)	124 (64.2%)	40 (58.8%)	
T4a	1 (0.6%)	1 (0.5%)	0 (0%)	
BRAF mutation				0.291
Absent (12.9%)	27 (15.5%)	22 (11.4%)	6 (8.8%)	
Present (87.1%)	147 (84.5%)	171 (88.6%)	62 (91.2%)	
Stimulation Tg				
Mean sTg (±SD), ng/ml	1.24 (±3.92)	2.50 (±7.79)	3.83 (±7.62)	0.001
<1 ng/ml	142 (81.6%)	145 (75.1%)	38 (55.9%)	<0.001
≥1 ng/ml	32 (18.4%)	48 (24.9%)	30 (44.1%)	

*Abbreviations*: *LN* lymph node, *Tg* thyroglobulin

**Fig. 2:**
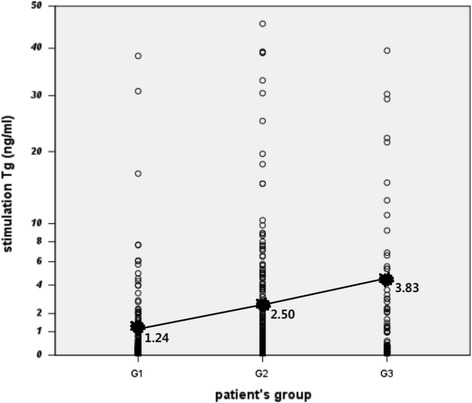
The distribution and mean value of the sTg level according to the patient’s risk groups. The sTg denoted a mean value which gradually increased for each patient’s risk group. (*G1* group I, *G2* group II, *G3* group III)

Among these factors, the remarkable factors included the mean numbers of mLNs and the sTg level. The mean number of mLNs increased from the group I to the group III. The mean sTg value gradually increased with the patient risk group and also produced a significant result when compared across groups at an sTg cutoff value of 1 (*p* < 0.001; Table 3, Fig. 2).

### Recurrence-free survival according to the stratified groups

**Fig. 3:**
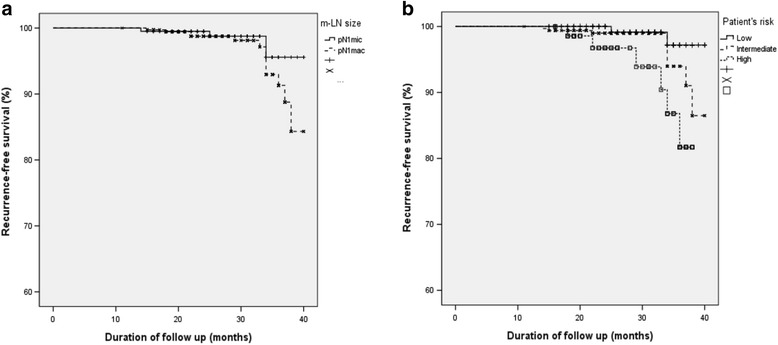
Recurrence-free survival according to the size of mLN (**a**) or the groups of patient’s risk (**b**) by Kaplan-Meier curves. **a** These showed poorer RFS in the pN1mac than pN1mic (3-year RFS 95.5% versus 92.7%, *p* = 0.136). **b** These showed better RFS in the risk group I than the other two groups (*p* = 0.015)

Fourteen cases of disease recurrence were identified during the median follow-up period of 28 months (range 14–40 months) across all patients. The mean time of recurrence was 27.1 months. When compared according to the size of mLN, the count of recurrence was observed in more of the pN1mac patients than the pN1mic patients (10/237, 4.2% versus 4/198, 2.0%, respectively). Also, the 3-year RFS was 95.5% in the pN1mic and 92.7% in the pN1mac, but this difference was not significant (*p* = 0.136). In contrast, recurrence-free survival was significantly different between the stratified risk groups; the highest relapse rate and the lowest 3-year RFS occurred in the patient group III (group I: 2/174, 1.2 and 97.1%; group II: 6/193, 3.1 and 94.5%; group III: 6/68, 8.8 and 85.6%; *p* = 0.015; Fig. 3).

## Discussion

In this study, we attempted to create a risk stratification for the 435 patients who underwent postoperative RAI ablation due to nodal metastasis with PTC, and we obtained significant results. The sTg values exhibited a tendency to increase with risk as defined by the stratification of the three risk groups based on the size of mLN and LNR. The RFS was significantly different between the three groups. Risk stratification based on the size of mLN and the LNR could be considered a useful method for the classification of postoperative patients with PTC. In other words, the patient risk groups I, II, and III in the current study may be referred to as low-, intermediate-, and high-risk groups.

The size of the primary tumor or the mLN, the number of mLNs, the presence of ETE, and the tumor stage have already been demonstrated to be factors that are associated in increased risk in previous studies [11–13].

Bardet et al. argued that modifications to the staging systems for the risk of recurrence of PTC are needed. This argument is supported by the finding that the 4-year risk of persistent/recurrent disease significantly differs according to size of mLN (pN1mac 49% versus pN1mic 24%, *p* = 0.03; pN0 12%, *p* = 0.01; or pNx 6%, *p* < 0.001). Moreover, the pN1mac was an independent predictor of persistent disease, but the pN1mic was not. Therefore, these parameters were described as pN1mic and pN1mac at quite different stages. However, this study did not mention any relations to the LNR [16]. A meta-analysis was published in 2012 and referred to risk stratifications of differentiated thyroid cancers that are based on the specific LN characteristics, such as the size of mLN and the number of m-LNs. Lower risk N1 disease was associated with conditions such as N0 clinical status, micrometastasis, and fewer than five m-LNs; these conditions predicted a recurrence rate below 5%. In contrast, higher risk N1 disease was associated with the conditions of N1 clinical status, macrometastasis greater than 3 cm, and a number of m-LNs greater than five; these conditions predicted a recurrence risk above 20% [11]. Moreover, Ricarte-Filho et al. attempted to stratify PTC patients with the cervical LNM using the number of mLMs, the oncogenic BRAF status, and the presence of ETE [13]. Although different methods have been used for stratification, these attempts at risk stratification can all be considered to have the goal of predicting the recurrence rates or prognoses of patients with PTC [11–13, 17]. In another attempt of the current study, the stratification based on the performance of MRND and the LNR did not exhibit meaningful results. Another three risk groups were made for 580 patients who underwent RAI ablation treatment following thyroid surgery including MRND, as follows: group I', MRND (-) with LNR ≤ 0.5; group II', MRND (-) with LNR > 0.5 or MRND (+) with LNR ≤ 0.5; and group III', MRND (+) with LNR > 0.5. The number of patients in the risk group III' was only 17 patients, they were too small patients compared to the other two groups. Also, the sTg value did not exhibit a gradually increasing pattern (group I': 0.98 ng/ml; group II': 3.12 ng/ml; group III': 2.83 ng/ml).

The prognosis and recurrence of PTC are related to the presence of LN metastasis or ETE, greater primary tumor size or a greater number of mLNs, and a greater LNR [2, 4, 17]. Interest in the size of mLN has increased in recent years, although little research has been conducted in this area. In the majority of studies, the sizes of mLN have been compared following the classification of the patients as having micrometastasis or macrometastasis. Consequently, macrometastasis has been confirmed as a predictor of recurrence and poorer prognosis [1, 16, 18]. As illustrated in Table 2, the presence of gETE, and the mLN ratio of >0.5 were significantly associated with macrometastasis. The other factors that were found to be associated with the size of mLN were not significantly different from the results of previous studies [5, 6, 18].

Regarding the LNR, the studies that have included only central LN metastases are quite numerous. The central LNR is an independent factor related to the recurrence of PTC, and the macrometastasis of a CLN is clinically meaningful as an independent variable during the follow-up after surgery [19, 20]. However, analyses that have included both of central and lateral LN metastases are scarce. Therefore, large and well-designed studies are required in the future.

Additionally, the LNR cutoff value is still under debated, and the definitions of this cutoff value vary considerably between institutions. Studies published in 2013 and in 2014 reported LNR cutoff values of 0.4 and 0.65, respectively [5, 19]. Our statistical analyses were performed after defining a cutoff value of 0.5 based on the ROC curve method. Consequently, the LNR and central LNR were among the factors that exhibited statistically significant differences according to the size of mLN and the patient risk group.

The serum Tg level has been reported to be among the useful predictors of postoperative recurrence or survival. During the follow-up period, a sudden increase in the serum Tg level could be indicative of disease recurrence [8, 21]. Moreover, postoperative stimulation Tg value was used in the decision to apply RAI ablation therapy following surgery. The cutoff value was not clear, but in general, a value of approximately 1 ng/ml is considered to indicate stable disease status [7, 22]. Among our results, the mean sTg value was observed to gradually increase with the three stratified risk groups, and 1.24 ng/ml was the mean sTg value of the risk group I. (Fig. 2). Therefore, we suggest that RAI ablation can be omitted for only in the group I, which means a low-risk group.

This study had several limitations. First, the follow-up period was not sufficiently long to obtain information about the mortality rates of the patients. Second, we were not able to analyze the recurrence rates according to distant metastases and locoregional recurrence due to the small number of recurrence events in the short follow-up period. Additional studies with longer follow-up times are needed in the future. Third, more than 160 patients were entirely excluded from the present study because BRAF mutation tests were not performed for several months in 2014. This issue may have resulted in selection bias.

Nevertheless, we used well-organized prospective data collection that resulted in a wealth of information, and the analyses were performed on the data from a relatively large number of patients. Our study is the first to create a patient risk stratification based on the size of mLN and LNR. The results of this study suggest that this stratification will be valuable for informing the decision to apply RAI ablation to postoperative patients.

## Conclusions

Macrometastases in the LNs and intermediate- and high-risk statuses were strongly correlated with increased sTg levels. Moreover, the highest RFS occurred in the low-risk patient group. The patient risk groups I, II, and III in the current study may be referred to as low-, intermediate-, and high-risk groups. Clinicians should consider the possibility of recurrence and should base the decisions regarding the application of RAI ablation on the size of mLN and the patients’ risk groups. We suggest to consider omitting the RAI ablation for patients in the low-risk group. In order to be sure, we suggest that more large-scale and well-designed studies and long-term follow up data is needed.
